# Investigating the epigenetic profile of the inflammatory gene *IL-6* in late-life depression

**DOI:** 10.1186/s12888-017-1515-8

**Published:** 2017-10-25

**Authors:** Joanne Ryan, Lauren Pilkington, Katharina Neuhaus, Karen Ritchie, Marie-Laure Ancelin, Richard Saffery

**Affiliations:** 1 Department of Epidemiology and Preventive Medicine, Monash University, Melbourne, VIC Australia; 2 Cancer & Disease Epigenetics, Murdoch Childrens Research Institute, Royal Children’s Hospital & Department of Paediatrics, University of Melbourne, Parkville, VIC 3052 Australia; 3 INSERM, Univ Montpellier, Neuropsychiatry: Epidemiological and Clinical Research, Montpellier, France

**Keywords:** *IL6*, DNA methylation, Epigenetics, Buccal, Late-life depression, Antidepressants, Inflammation

## Abstract

**Background:**

It is well established that there is a link between inflammation and depression, with several studies reporting increased circulating levels of the pro-inflammatory cytokine, interleukin-6 (*IL6*), in depressed individuals. Peripheral epigenetic marks, including DNA methylation, hold promise as biomarkers for a range of complex conditions, with potential to inform diagnosis and tailor interventions. The aim of this study was to determine whether individuals with depression display differential methylation of the *IL6* gene promoter compared to individuals without depression.

**Methods:**

The ESPRIT study of later life neuropsychiatric disorders used a random sampling framework to select non-institutionalised participants aged ≥65 years and over living in the Montpellier region of France. Major depressive disorder (MDD) was assessed using the Mini International Neuropsychiatric Interview (MINI) according to DSM-IV criteria. High levels of depressive symptoms were defined as a score of ≥16 on the Centre for Epidemiologic Studies Depression Scale (CES-D). *IL6* promoter DNA methylation was measured on a sub-sample of 380 participants who provided buccal samples.

**Results:**

Individuals with depression (current MDD or high depressive symptoms) had lower *IL6* methylation levels at one of the four sites investigated, however the effect size was small (∆ 2.4%, SE 0.009, *p* = 0.006). Interestingly, antidepressant use was independently associated with higher *IL-6* methylation at the same site (∆ 4.6%, SE 0.019, *p* = 0.015). In multivariate linear regression analyses adjusting for covariates, including sex and smoking status, these associations remained. There was no effect modification when considering *IL6* genotype.

**Conclusion:**

This study presents evidence that *IL6* methylation may be a marker of depression status in older individuals, however further work is now needed to replicate these findings and to assess the association with inflammatory status of individuals.

## Background

Depression in the elderly is common and often a chronic medical illness with high disease burden [1]. It is associated with heightened comorbidity [2] and an increased risk of mortality [3]. Unfortunately, despite the significant burden, depression in later life is underdiagnosed [1] and when treatments are given, they are often ineffective [4]. Accurate and timely diagnosis thus remains an important priority which could lead to reductions in health burden and suffering.

Inflammation has long been thought to play an important role in depression. A heightened inflammatory response is commonly observed in patients with major depressive disorder, with increased levels of inflammatory markers in both central and peripheral tissue [5]. Cytokine treatments have been linked to the occurrence of new depressive episodes [6] and individuals with major depression have a high frequency of comorbid chronic inflammatory diseases [7]. Adding to this, pro-inflammatory cytokines can induce changes in the levels of neurotransmitters and dysregulation of neuroendocrine function [8], which are commonly recognised features of depression [9]. This suggests that inflammation may be involved in depression pathogenesis.

Interleukin-6 (IL6) is a pro-inflammatory cytokine and is expressed in neurons and glia cells [10]. It plays a major role during the immune response and acute-phase reaction [11]. IL6 is one of the most consistent inflammatory markers elevated in MDD [12–15]. A recent meta-analysis of 18 studies confirmed this finding, with significantly higher IL6 blood levels in individuals with MDD compared to healthy controls [16].

There are several broad lines of evidence to suggest the involvement of epigenetic processes in psychiatric disorders, including depression [17]. Epigenetic marks are also increasingly recognised for their potential as peripheral biomarkers of disease status and risk prediction [18]. Indeed, a number of recent studies have reported that genes known to be implicated in depression, such as *SLC6A4*, *NR3C1* and *BDNF*, are differentially methylated in peripheral blood and/or buccal cells of individuals with depression, compared to those without [19–21]. However, no study has yet investigated whether inflammatory genes are differentially methylated in peripheral tissues of individuals with depression.

## Methods

### Aim of the study

The aim of our study was therefore to determine whether promoter DNA methylation of *IL6* in peripheral tissue was associated with depression in the elderly. Given that increased IL6 protein levels have been reported in MDD and that promoter gene methylation is commonly associated with reduced gene transcription, we hypothesised that depression would be associated with decreased *IL6* DNA methylation. Secondary aims of our study were to determine whether the use of antidepressant treatment modifies *IL6* methylation and to investigate the impact of a common *IL6* genetic variant on these associations.

### Design & setting of the study

The 380 participants included in this study were part of the larger ESPRIT study of neuropsychiatric disorders in France [22]. Participants in ESPRIT were randomly recruited from the electrical rolls within the Montpellier region, with inclusion criteria requiring that they were living in the community and aged 65 years or older. After providing written informed consent, participants responded to a number of standardized questionnaires administered during face-to-face interviews. Clinical assessments were also performed. The study has been approved by the Ethical Committee of University Hospital of Kremlin-Bicêtre.

### Depression diagnosis

At recruitment and each wave of follow-up, major depressive disorder (MDD) was diagnosed using the Mini International Neuropsychiatric Interview (MINI) according to the Diagnostic and Statistical Manual of Mental Disorders-IV (DSM-IV) criteria [23]. The medical history and medication use of all suspected cases were reviewed to validate the diagnosis. Depressive symptoms were assessed at each wave using the Centre for Epidemiologic Studies – Depression scale (CES-D), a commonly used tool to assess depression and which has been validated in the elderly population [24, 25]. A score of 16 or more on the CES-D is considered indicative of depressive symptoms warranting further clinical investigation. Depression was thus defined in this study as either a high score on the CES-D (≥16) or a current diagnosis of MDD [26].

### DNA extraction & genotyping

Buccal swabs were provided by participants around the forth wave of follow-up in the ESPRIT study [19]. DNA was extracted using standard methods [27]. We genotyped the functional variant *rs1800795* of *IL6* on chromosome 7 given that this is the most widely investigated SNP within the promoter region of the *IL6* gene, often referred to as “-174”. The variant C allele has been shown to result in lower IL6 cytokine levels than that of the wild-type G allele, in various populations [28–30]. This SNP has also been associated with a number of other inflammatory-related health conditions including arthritis [28], diabetes [31], and dementia [32]. Preliminary evidence suggests that this SNP may even influence *IL6* expression in the frontal cortex [33]. Genotyping of *rs1800795* was performed by KBiosciences (Middlesex, UK) using the KBioscience Competitive Allele-Specific Polymerase Chain Reaction (PCR) SNP genotyping system (KASPar) (as described previously [34]). PCR products were scanned using the BMG Labtech Pheraster scanner and the results were interpreted with KBioscience KlusterCaller 1.1 software. The Hardy-Weinberg equilibrium (HWE) was calculated using a chi-squared test by comparing the actual and predicted genotype frequencies.

### Methylation analysis

Bisulphite conversion of 500 ng of DNA was performed using the EZ-96 DNA Methylation-Lightning™ MagPrep according to the manufacturer instructions (Irvin, CA, USA). The Epidesigner software (http://www.epidesigner.com/) was used to help design the methylation assay, which targeted the CpG island in the promoter region of the gene. This is an area which has been shown to be differentially methylated and associated with other phenotypes in previous studies [35, 36]. The forward (5′-aggaagagagTAGGATTGGAGATGTTTGAGGTTTA) and reverse (5′-cagtaatacgactcactatagggagaaggctAACAACACAACTAAAAACCTACCTCT) primers amplified a 234 bp region of the gene in bisulphite converted DNA commencing at position chr7:22,726,201 in exon 1 on the UCSC Genome Browser (GRCh37/h19 assembly).

*IL6* methylation at 4 CpG units across 7 CpG sites was quantified using the SEQUENOM MassARRAY analysis system (San Diego, CA, USA) [37] and the EpiTyper software (v.1.2; SEQUENOM). All samples were amplified in triplicate, given that this is the most variable step in methylation analysis, and all replicate amplification products were used to generate methylation data [38]. The mean methylation, expressed at each CpG unit as the percentage of methylated cytosine over the sum of methylated and unmethylated cytosine, was calculated from the three technical replicates. Outliers which differed by more than 10% from the median value were discarded, and the mean methylation of the remaining two or three values was calculated. Methylation data was generated for 380 participant samples and these were considered broadly representative of the full ESPRIT sample with no significant differences in terms of depression status, age, sex or health factors (*p* > 0.05 for all comparisons).

### Statistical analysis

All statistical analysis was performed using Stata version 14.1 (StataCorp, Texas, USA). Univariate analysis was performed to firstly investigate unadjusted associations between *IL6* methylation and depression status. Multivariate linear regression analysis was then performed incorporating potential confounding factors, including antidepressant use. We also investigated the potential for independent and interacting effects of *rs1800795* on these associations.

## Results

Of the 380 participants, 92 (24.2%) had high depressive symptoms (CES-D ≥ 16) and 11 of these individuals also had a current diagnosis of MDD. The characteristics of the study population according to their depression status are shown in Table 1. Depressed participants were significantly more likely to be female, have a lower education level, live alone, and have a higher frequency of cognitive impairment. They were also significantly more likely to use antidepressants (*p* < 0.0001). These characteristics were considered as potential cofounders which could influence the association between depression and *IL6* methylation.

**Table 1:** Participant characteristics according to depression status

Characteristic	No depression *n* = 288	MDD and/or CES-D ≥ 16 *n* = 92	Difference *p*-value^a^
	Mean ± SD		
Age	71.6 ± 4.5	71.6 ± 4.2	0.987
	%		
Female	52.4	78.3	< 0.001
Higher (≥12 years) education level	39.6	25.0	0.011
Living alone	18.4	38.0	< 0.001
Regular alcohol consumption (>24 g/day)	22.6	15.2	0.141
Current smoking (>10 pack years)	37.5	32.6	0.421
Functional impairment^b^	1.4	4.3	0.085
Cognitive impairment^c^	3.5	14.1	< 0.001
Cardiovascular disease^d^	12.5	7.6	0.197
Other reported health conditions or related treatments^e^	13.9	8.7	0.192
Obese (BMI ≥ 30)	6.3	10.9	0.140
Antidepressant use^f^	1.7	10.9	< 0.001

^a^Calculated from a chi squared test, except for age where a t-test was used

^b^Unable to independently complete ≥2 items on the Instrumental Activities of Daily Living, or the Activities of Daily Living scales

^c^Mini-Mental State Examination score < 24

^d^Angina pectoris, myocardial infarction, stroke, cardiovascular surgery, arteritis

^e^Hypertension, high cholesterol, diabetes, thyroid problems, asthma, recent cancer diagnosis or cardiovascular disease

^f^According to the World Health Organisation’s ATC classification, with just over half being selective serotonin receptor inhibitors (SSRIs)

**Fig. 1:**
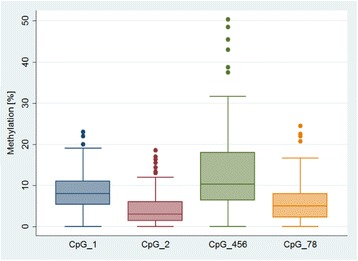
Distribution of *IL6* methylation levels at each of the 4 CpG units

The *IL6* assay amplified a 234 bp region of the gene promoter and methylation could be measured at four CpG units, two of which comprised multiple individual CpGs (CpG 4.5.6 and CpG 7.8). Overall the mean level of methylation was relatively low, with CpG 4.5.6 being the highest and most variable (Fig. 1).

**Fig. 2:**
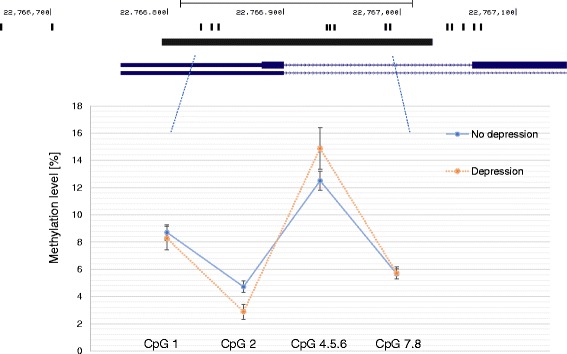
*IL6* methylation assay and mean methylation levels in depressed (n = 92) and non-depressed (n = 288) participants. Footnote: Black bar indicates the methylation assay and position on chromosome 7 is given. Black bands are the CpG sites (numbered from left to right). Error bar on the graph indicate the standard deviation

In unadjusted analysis we found evidence that depressed individuals had lower *IL6* methylation at CpG 2 (2.88% versus 4.72%, *p* = 0.033) (Fig. 2). In contrast, depressed individuals had a higher mean *IL6* methylation level at CpG 4.5.6 compared to non-depressed participants, but this difference was not significant (14.88% versus 12.50%, *p* = 0.11). There was no difference between depressed and non-depressed groups at CpG 1 or CpG 7.8 (*p* < 0.20).

We next investigated whether any of the participant characteristics were also associated with *IL6* methylation at CpG 2, and a non-significant trend was observed with antidepressant use. Interestingly, this was in the reverse direction to that for depression (Table 2). When depression status and antidepressant use were combined together in a model, the effect sizes increased further. Depression was associated with a 2.4% decreased *IL6* methylation while antidepressant use was associated with a mean 4.6% increase in methylation. These associations remained after adjustment for other potential confounding factors such as sex, living status and cognitive impairment (Table 2), as well as others described in Table 1.

**Table 2:** Linear regression models for the association between depression and antidepressant use with *IL6* methylation at CpG 2

Characteristics	Unadjusted associations^a^		Adjusted associations		Multivariate adjusted model	
	β (SE)	*p*	β (SE)	*p*	β (SE)	*p*
Depression	−0.018 (0.009)	0.033	−0.024 (0.009)	0.006	−0.028 (0.009)	0.003
Antidepressants	0.032 (0.019)	0.090	0.046 (0.019)	0.015	0.046 (0.019)	0.017
Female Sex					0.003 (0.008)	0.71
Living alone					0.010 (0.010)	0.29
Cognitive impairment					0.004 (0.019)	0.83

*β* the beta coefficient from the linear regression model, *SE* standard error

^a^Depression and antidepressant use considered independently in separate models

For 365 of the 380 participants in the study, *rs1800795* genotype data was available. The frequencies of genotypes were as follows: GG 35.6%, GC 48.8%, CC 15.6%. These were in Hardy-Weinberg Equilibrium (chi-squared 0.093, *p* = 0.76). Genotype frequencies were not significantly different between the depressed and non-depressed groups (*p* = 0.50). Furthermore, *rs1800795* was not associated with *IL6* methylation at any of the four CpG units examined (*p* > 0.14 for all units).

## Discussion

It is widely established that inflammation is implicated in depression, although the exact direction of the association is unclear. Inflammation is thought to play a role in the pathogenesis of depression, but depression itself may result in increased inflammation. Inflammation and depression may also have similar aetiological underpinnings such as chronic stress [39]. IL6 appears to be one of the most robust inflammatory markers associated with depression [40], and elevated levels have been observed in older individuals with new depressive episodes. Increased IL6 have also been associated with reduced grey matter in the brain, often seen in depression [41].

Drawing on data and biospecimens gathered from a prospective study of psychiatric disorders, we investigated whether *IL6* methylation in a peripheral tissue is associated with depression status. We found that individuals with depression had a significantly lower methylation at one CpG unit compared to non-depresesd elderly, while antidepressant use was associated with increased methylation at this same site. The commonly studied *rs1800795* genetic variant, linked to *IL6* expression and cytokine levels in the frontal cortex [33], was not associated with either *IL6* methylation, nor with depression status in this elderly sample.

Epigenetic mechanisms such as DNA methylation, can regulate gene expression, and are increasingly recognised for their involvement in a range of diseases, including depression [42]. Promoter DNA methylation of a number of candidate genes could also be a biomarker of disease status when measured in peripheral tissue [18]. This is the first study to investigate whether *IL6* methylation in a readily accessible tissue is associated with depression status, however methylation has been associated with a range of other conditions including Alzheimer’s dementia [43], prostate cancer [44] and obesity [45]. A recent study of 550 older men (mean age 73 years) examined the correlation between *IL6* methylation (at 2 CpG sites) and performance on tests of psychological distress (measured with a brief symptom inventory) and life satisfaction, but they failed to find any significant associations [46]. Our findings are in accord with our previous observations concerning brain-derived neurotrophic factor (*BDNF*) in the ESPRIT population; with increased methylation observed in individuals with depression [19]. Indeed, *BDNF* and *IL6* appear to have opposing roles in depression - BDNF is thought to interact negatively with inflammatory processes in the brain and there is attenuation of BDNF availability with increased inflammation [47].

We do not know the exact functional significance of the methylation changes observed in our study, given that we had no measure of *IL6* gene expression or circulating levels of IL6. Promoter DNA hypomethylation generally leads to increased gene expression and thus elevated protein levels and the region examined has previously been shown to bind a range of different transcription factors (ENCODE; data not shown). Indeed, a previous study of 33 adults with a lifetime history of depression (assessed with the 9-item Patient Health Questionnaire), reported that *IL6* methylation was inversely associated with circulating IL6 (and C-reactive protein), but only for individuals with lifetime depression [48]. They found no association in non-depressed individuals and they did not examine whether methylation was different between depressed and non-depressed individuals. Furthermore, a recent small study of 50 MDD patients and 50 controls reported higher *IL6* mRNA expression in blood from MDD patients [33]. This is in line with our association regarding decreased *IL6* methylation in depression.

The findings of our analysis cannot draw any conclusions regarding cause and effect. DNA methylation patterns are tissue specific, but depression is considered a systemic disease [49]. It is possible that *IL6* methylation is reflective or results from processes occurring with depression, it could influence cytokine levels themselves and thus directly influence depression risk, or it could be a marker of a common aetiology. Our finding that antidepressant use had opposing associations with *IL6* methylation compared to that of depression is particularly interesting, given the previous observations that antidepressants are effective in reducing elevated IL6 levels in MDD [50].

Our analysis involved a sub-sample of individuals from the ESPRIT study who were recruited from the general population. This recruitment increases the generalisability of findings compared to case-control studies involving depressed patients. We have demonstrated that the participants included in this analysis were not significantly different from the full ESPRIT cohort in terms of the general characteristics examined, as well as depression prevalence and frequency of antidepressant use. However, the overall prevalence of current MDD in our sample was relatively low, as might be expected for a community-based sample, and we were not able to investigate this separately. Furthermore, despite involving only a sub-sample of ESPRIT participants, the size of the sample is still considerable, compared with other studies in the broader field.

Limitations to the analysis include the cross-sectional study design, focus on a single tissue, and a lack of direct measures of inflammation or infection. It is possible that the associations we observed where driven, at least in part by differences in the levels of cytokines or in the cellular composition of the buccal swabs between individuals with and without depression. Buccal swabs typically contain a major proportion of buccal epithelial cells, but also a proportion of leukocytes, and these could differ between individuals and according to depression status. We investigated only one *IL6* genetic variant, which was not found to be associated with methylation, nor to modify the association between methylation and depression. Given the now recognised importance of underlying genetic variation in influencing DNA methylation [51], it seems likely that other *IL6* variants might be associated with methylation in this region. However, *rs1800795* is a recognised functional variant and has been associated with IL6 protein levels, at least in some populations [28–30].

## Conclusion

This study provides evidence that both depression and antidepressant use may be associated with altered *IL6* DNA methylation in buccal epithelia cells. While no claims regarding causality can be made, the observation that depression and antidepressants have opposing associations is intriguing and worthy of further investigation. In particular, it would be interesting to measure *IL6* methylation in longitudinal samples collected at multiple time-points from large cohorts including depressed individuals and those on treatment. This would enable the investigation of whether methylation patterns track with disease incidence and remission, both in the presence and absence of antidepressant treatment.

## Abbreviations

CES-D: Centre for Epidemiological Studies Depression Scale
IL6: Interleukin 6
MDD: Major depressive disorder
